# Supplementary material for: A self-powered intracardiac pacemaker in swine model
Source: Nat Commun. 2024 Jan 13;15:507. doi: 10.1038/s41467-023-44510-6 (PMC10787765; doi:10.1038/s41467-023-44510-6)
Supplement: Supplementary file 2 — Supplementary information [file 41467_2023_44510_MOESM2_ESM.pdf]

Supplementary information

A self-powered intracardiac pacemaker in swine model

Zhuo Liu[1,2†], Yiran Hu[3,4†], Xuecheng Qu[1,5†], Ying Liu[1,5†], Sijing Cheng[3], Zhengmin Zhang[6], Yizhu Shan[1], Ruizeng Luo[1], Sixian Weng[3], Hui Li[7], Hongxia Niu[3], Min Gu[3], Yan Yao[8], Bojing Shi[1,2], Ningning Wang[6*], Wei Hua[3*], Zhou Li[1,5*], Zhong Lin Wang[1,9]

[1]Beijing Key Laboratory of Micro-nano Energy and Sensor, Beijing Institute of Nanoenergy and Nanosystems, Chinese Academy of Sciences, Beijing 101400, China.

[2]Key Laboratory of Biomechanics and Mechanobiology, Ministry of Education, Beijing Advanced Innovation Center for Biomedical Engineering, School of Engineering Medicine, Beihang University, Beijing 100191, China.

[3]Department of Cardiology, The Cardiac Arrhythmia Center, State Key Laboratory of Cardiovascular Disease, National Clinical Research Center of Cardiovascular Diseases, Fuwai Hospital, National Center for Cardiovascular Diseases, Chinese Academy of Medical Sciences and Peking Union Medical College, Beijing, 100037, China.

[4]Department of Cardiology and Macrovascular Disease, Beijing Tiantan Hospital, Capital Medical University, Beijing 100070, China.

[5]School of Nanoscience and Technology, University of Chinese Academy of Sciences, Beijing 100049, China.

[6]School of Electronics and Information, Hangzhou Dianzi University, Hangzhou, 310018, China.

[7]Department of Ultrasound, State Key Laboratory of Cardiovascular Disease, National Clinical Research Center of Cardiovascular Diseases, Fuwai Hospital, National Center for Cardiovascular Diseases, Chinese Academy of Medical Sciences and Peking Union Medical College, Beijing, 100037, China.

[8]Department of Cardiology, Beijing Anzhen Hospital, Capital Medical University, Beijing 100029, China.

[9]Georgia Institute of Technology, Atlanta, GA 30332–0245, USA

*Corresponding author. Email: ning.wang@hdu.edu.cn (N. Wang)

drhuawei@fuwai.com (W. H.); zli@binn.cas.cn (Z. Li)

[†]These authors contributed equally to this work.

Contents

Supplementary Note 1. Energy output of SICP during each cardiac motion cycle

Supplementary Note 2. Maximal Power output of SICP

Supplementary Table 1. Comparison of representative commercial leadless pacemakers.

Supplementary Fig.1|The surface of SICP before and after deposited by Parylene-C

Supplementary Fig.2|SEM images of the surface of POM and PTFE without treatment by ICP

Supplementary Fig.3|Schematic illustration of EHU when working under different tilt angles

Supplementary Fig.4|A photograph of SICP fixed on the endocardium of the right ventricle in isolated heart

Supplementary Fig.5|V_{oc}, I_{sc} and power density of EHU with different resistance

Supplementary Fig.6|Stability and durability tests of EHU

Supplementary Fig.7|V_{oc}, I_{sc} and Q_{sc} of EHU at high frequency operation (~6.5 Hz)

Supplementary Fig.8|The PM powered by a capacitor with a capacity of 47 μF

Supplementary Fig.9|The cytoskeletal structures and cell nucleus of L929 cells stained by immunofluorescence at day 1, 2, and 3, respectively

Supplementary Fig.10|The viability of L929 cells on the encapsulation film tested by the Cell Counting Kit-8

Supplementary Fig.11|Localized tissues of the skin to deep layer muscle from the implantation location of the materials after 3 months implantation stained by Hematoxylin and Eosin (H&E)

Supplementary Fig.12|Hemolysis and coagulation test for the encapsulation materials

Supplementary Fig.13|A Photograph of the external jugular vein exposed with a small incision

Supplementary Fig.14|Photographs of delivery system, delivery catheter with SICP advancement, and Sutured incision after device implantation

Supplementary Fig.15|The schematic diagram of SICP implantation process

Supplementary Fig.16|The voltage of a capacitor from 0 V to 3 V within 9000 s at the same electrical output of SICP *in vivo*

Supplementary Fig.17| Photographs of the postoperative animal and statistical analysis of animal experiments.

Supplementary Note1. Energy output of SICP during each cardiac motion cycle

SICP coverts biomechanical energy from cardiac motion to electricity with time-dependent. The average output power $\bar{P}$ was related to the load resistance. The maximum energy output per cycle of SICP can be derived by the following equation [1]:

$$E = \bar{P}T = \int_0^T VIdt = \int_{t=0}^{t=T} VdQ = \oint VdQ$$

$$E_{max} = \frac{1}{2} Q_{sc,max}(V_{OC,max} - V_{OC,min})$$

$$E_{max} = \frac{1}{2} Q_{SC,max}\Delta V$$

Here, E_{max} represents the maximal output energy per cycle. The $Q_{SC,max}$ and ΔV of SICP in vivo were about 6.0 V and 8.5 nC, respectively. Therefore, the E_{max} of SICP for per cycle is about 0.026 μJ.

The pacing threshold energy can be derived by the following equation:

$$E_t = \int_0^T V_t \times Idt = V_t \times I \times T = \frac{V_t^2 \times T}{R}$$

Here, E_t represents the pacing threshold energy, V_t is the pacing threshold voltage. R represents the pacing resistance. T stands for stimulus pulse durations.

The mean pacing threshold voltage of SICP is 1.5 V with a pulse width of 0.5 ms, the mean pacing impedance of swine is about 953 Ω[2]. Therefore, the mean pacing threshold energy of SICP is 1.18 μJ in animal experiment. On the other hand, Ritter, P. et al. reported early performance clinical test of a miniaturized leadless cardiac pacemaker - Medtronic's Micra TPS[3]. The mean pacing capture threshold at the 3-month visit for the 60 patients measured with a pulse width of 0.24 ms was 0.51 V (95% CI, 0.45–0.56; P<0.0001), meeting the efficacy objective. Among these 60 patients, the mean electrical values for R-wave sensing amplitude, pacing impedance, and pacing capture threshold at a pulse width of 0.24 ms were as follows respectively: 11.7±4.5 mV, 719 ± 226 ohm, 0.57±0.31 V at implant, 15.6±4.8 mV, 662 ± 133 ohm, 0.48 ± 0.21 V at 1-month, and 16.1 ± 5.2 mV, 651 ± 130 ohm, 0.51 ± 0.22 V at 3-months.

$$E_{maximum\ pacing\ threshold} = (0.88\ V)^2 \times 0.24\ \text{ms} \div 493\ \Omega = 0.377\ \mu\text{J}$$

$$E_{meanmum\ pacing\ threshold} = (0.51\ V)^2 \times 0.24\ \text{ms} \div 719\ \Omega = 0.087\ \mu\text{J}$$

$$E_{minimum\ pacing\ threshold} = (0.26\ V)^2 \times 0.24\ \text{ms} \div 945\ \Omega = 0.017\ \mu\text{J}$$

Therefore, based on the rough calculation we can draw the following conclusion:

$$E_{minimum\ pacing\ threshold} < E_{max} = 0.026\ \mu\text{J} = 1/3.3$$

$$E_{meanmum\ pacing\ threshold} = 1/14.5\ \ E_{maximum\ pacing\ threshold}$$

Supplementary Note2. Maximal Power output of SICP

$$P_{max} = \frac{E_{max}}{T} = E_{max} f$$

$$P_{max} = \frac{1}{2} Q_{SC,max} \Delta V f$$

Here, P_{max} is the maximal power output of SICP. f denotes the operating frequency that drove by heart, which is about 1.5 Hz. Therefore, the P_{max} of SICP is about 0.039 μW.

Supplementary Table 1. Comparison of representative commercial leadless pacemakers.

Leadless Pacemaker	SICP	Nanostim LCP	Aveir LSP202V	Micra TPS
**Self-powered**	Yes	No	No	No
**Cost**	Low	High	High	High
**Length (mm)**	42	42	38	25.9
**Diameter (mm)**	6.8	5.99	6.5	6.7
**Volume (cc)**	1.52	1	1.1	0.8
**Mass (g)**	1.75	2	2.4	2
**Fixation Mechanism**	Helix/Hook	Helix	Helix	Hook
**MRI compatibility**	Yes	Yes	Yes	Yes

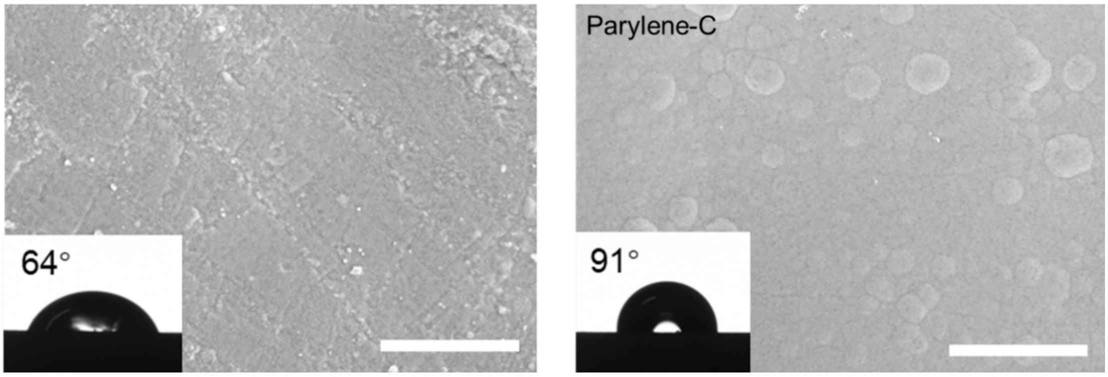

Supplementary Fig.1|The surface of SICP before and after deposited by Parylene-C (Scale bar = 10 μm).

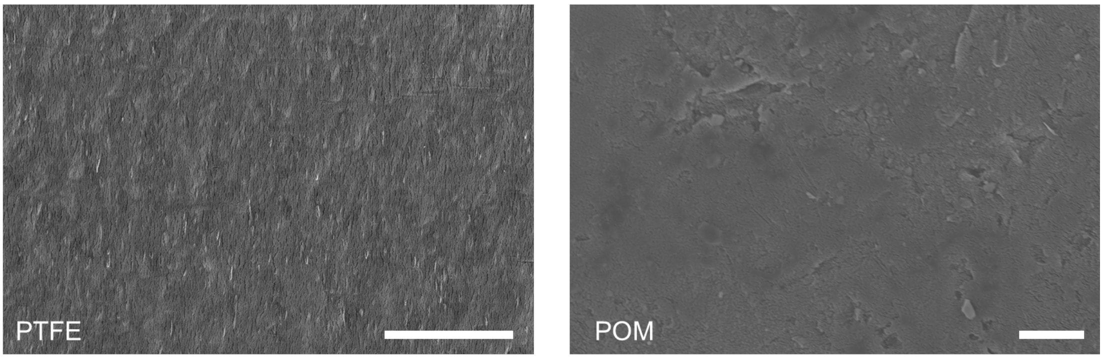

Supplementary Fig.2|SEM images of the surface of POM and PTFE without treatment by ICP (Scale bar = 5 μm)

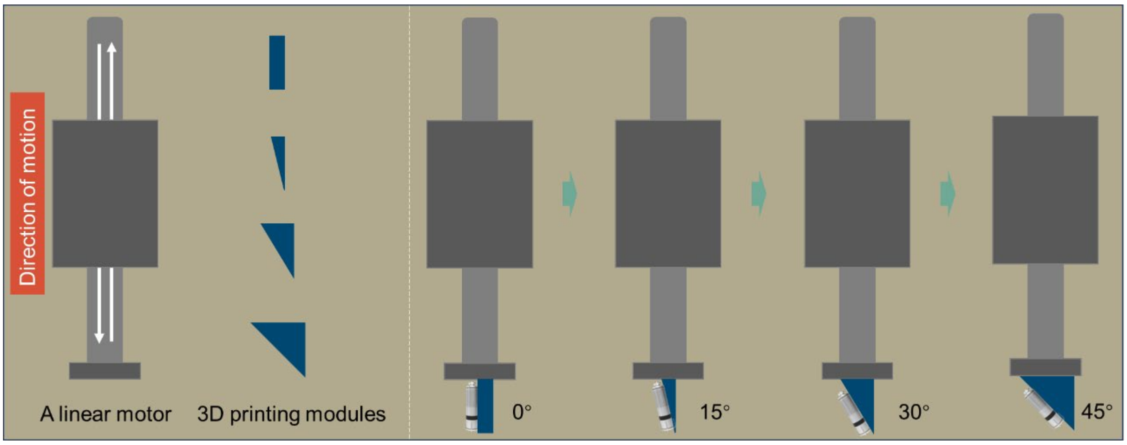

Supplementary Fig.3| Schematic illustration of EHU when working under different tilt angles.

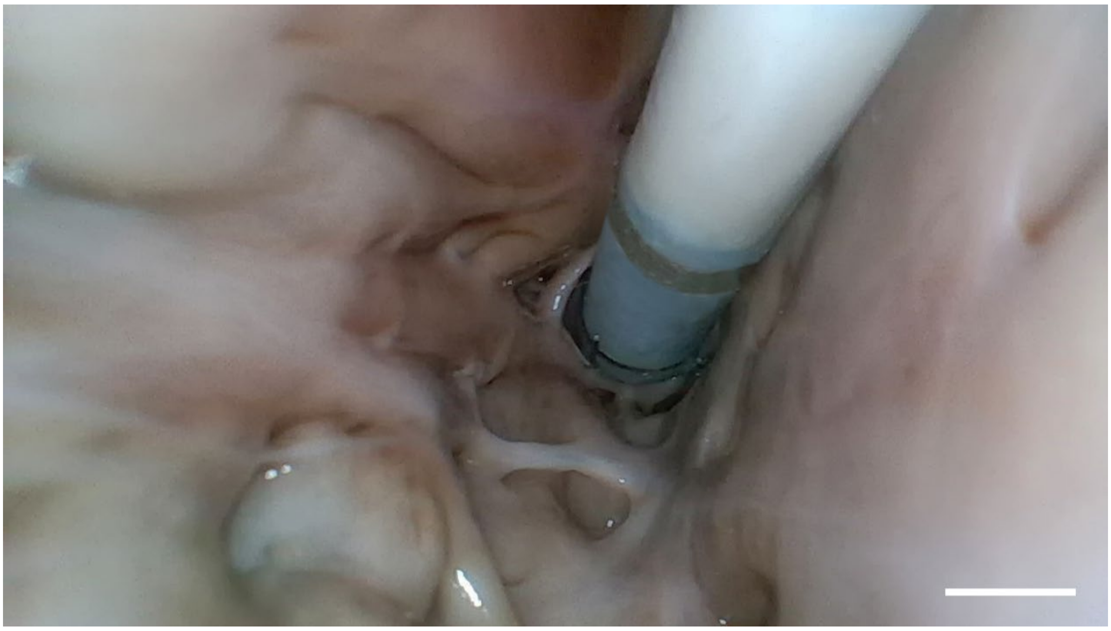

Supplementary Fig.4| A photograph of SICP fixed on the endocardium of the right ventricle in isolated heart (Scale bar = 0.5 cm).

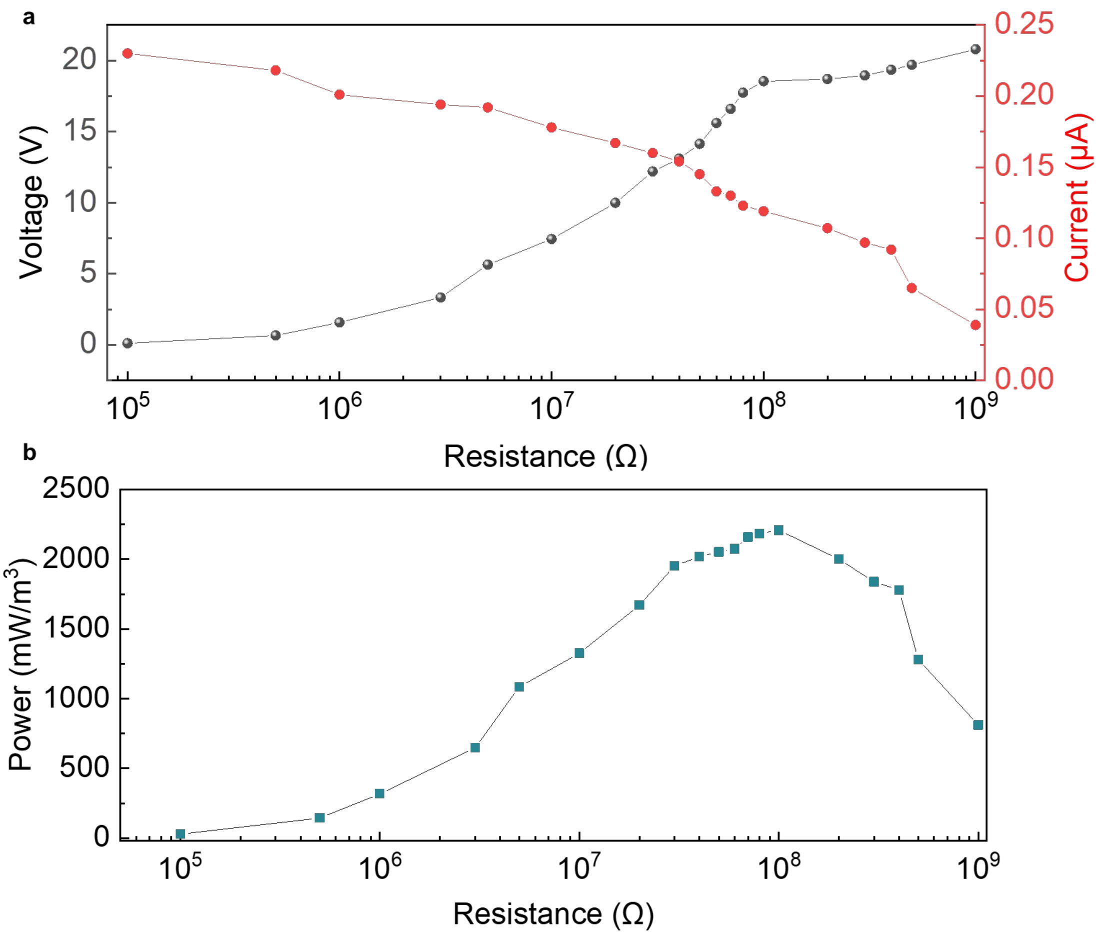

Supplementary Fig.5| a, V_{oc}, I_{sc} and (**b**) power density of EHU with different resistance. Source data are provided as a Source Data file.

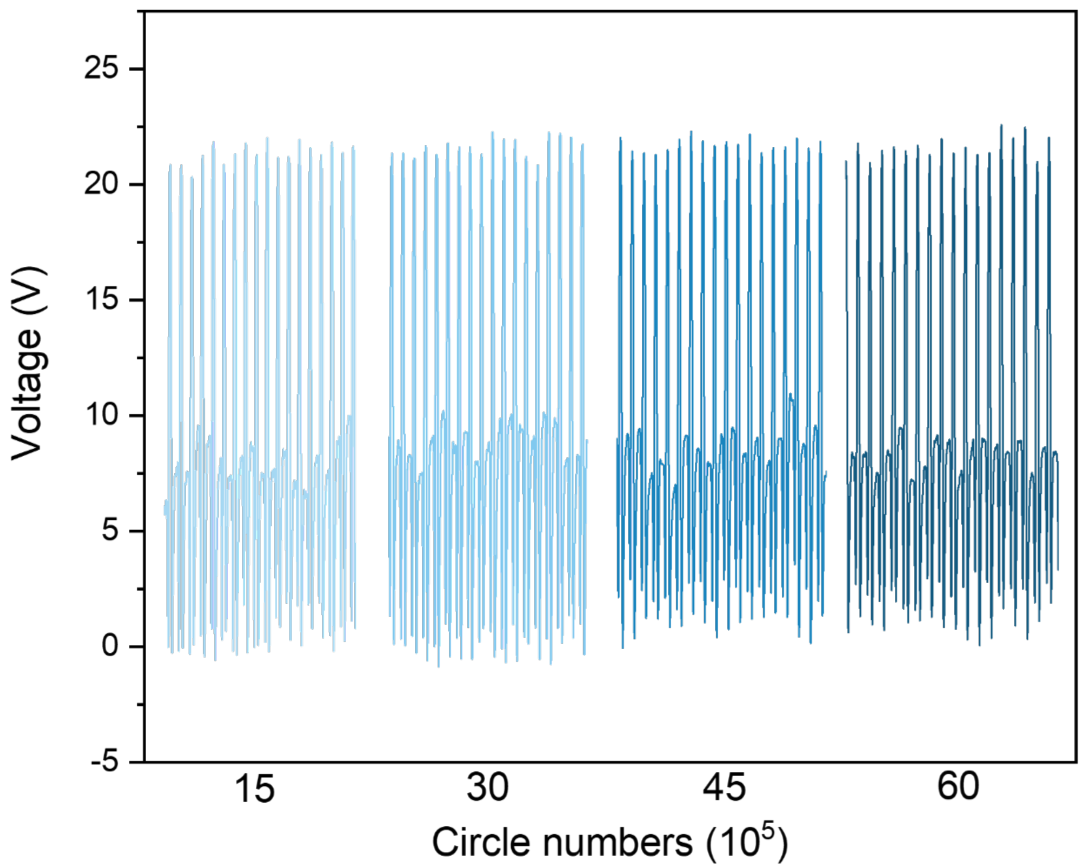

Supplementary Fig.6| Stability and durability tests of EHU. Source data are provided as a Source Data file.

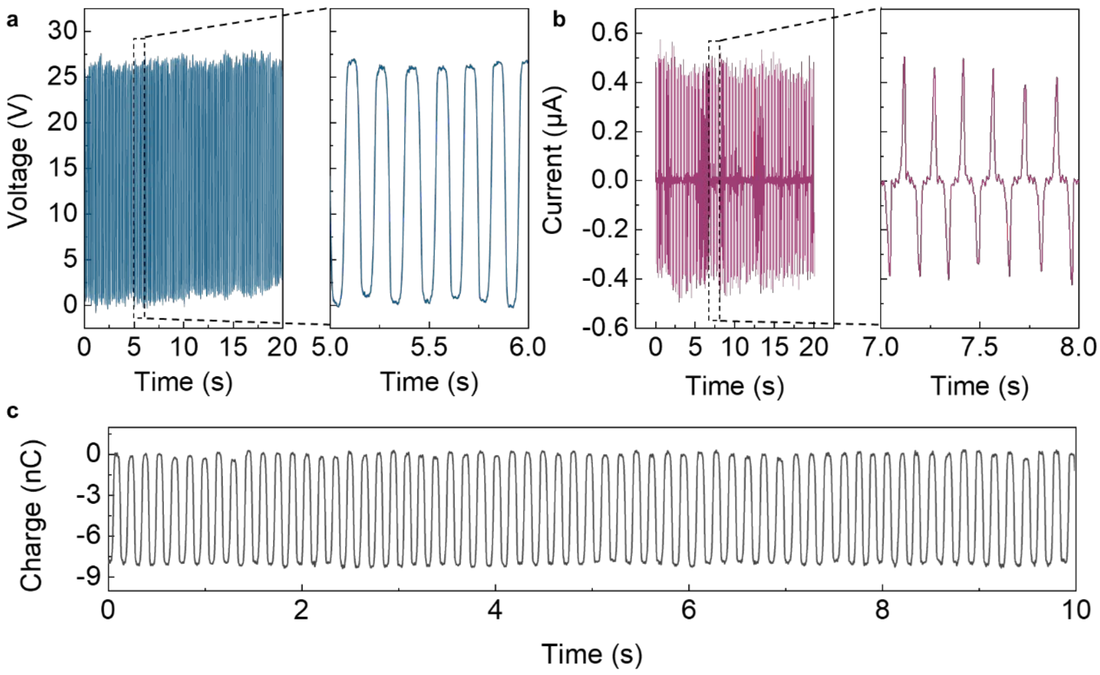

Supplementary Fig.7| a, V_{oc}, (**b**) I_{sc} and (**c**) Q_{sc} of EHU at high frequency operation (~6.5 Hz). Source data are provided as a Source Data file.

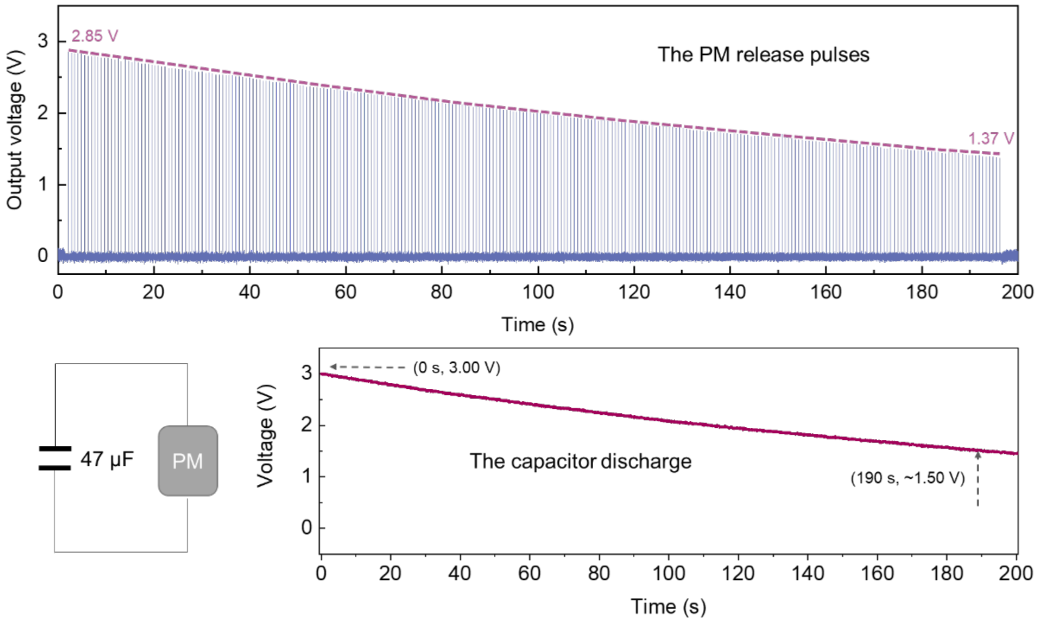

Supplementary Fig.8| The PM powered by a capacitor with a capacity of 47 μF. Source data are provided as a Source Data file.

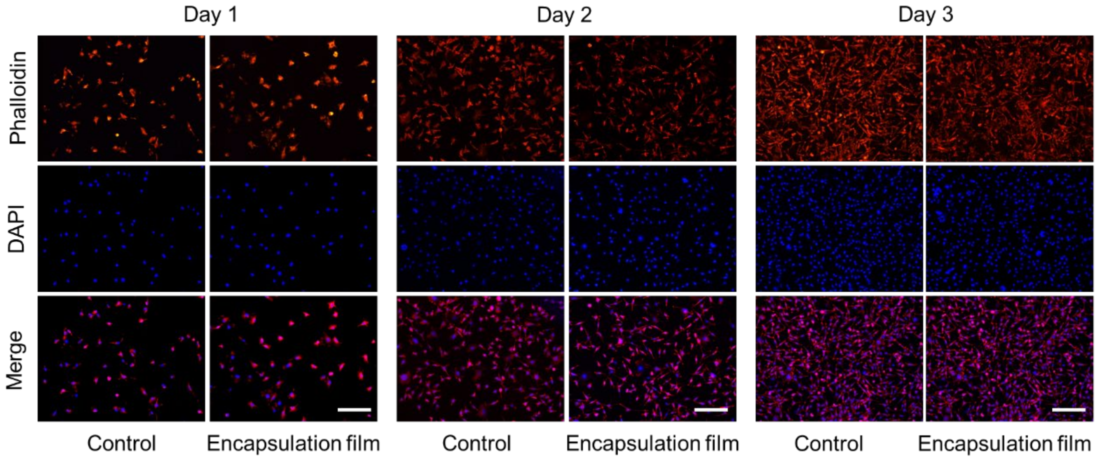

Supplementary Fig.9|The cytoskeletal structures and cell nucleus of L929 cells stained by immunofluorescence at day 1, 2, and 3, respectively. (Scale bar=200 μm)

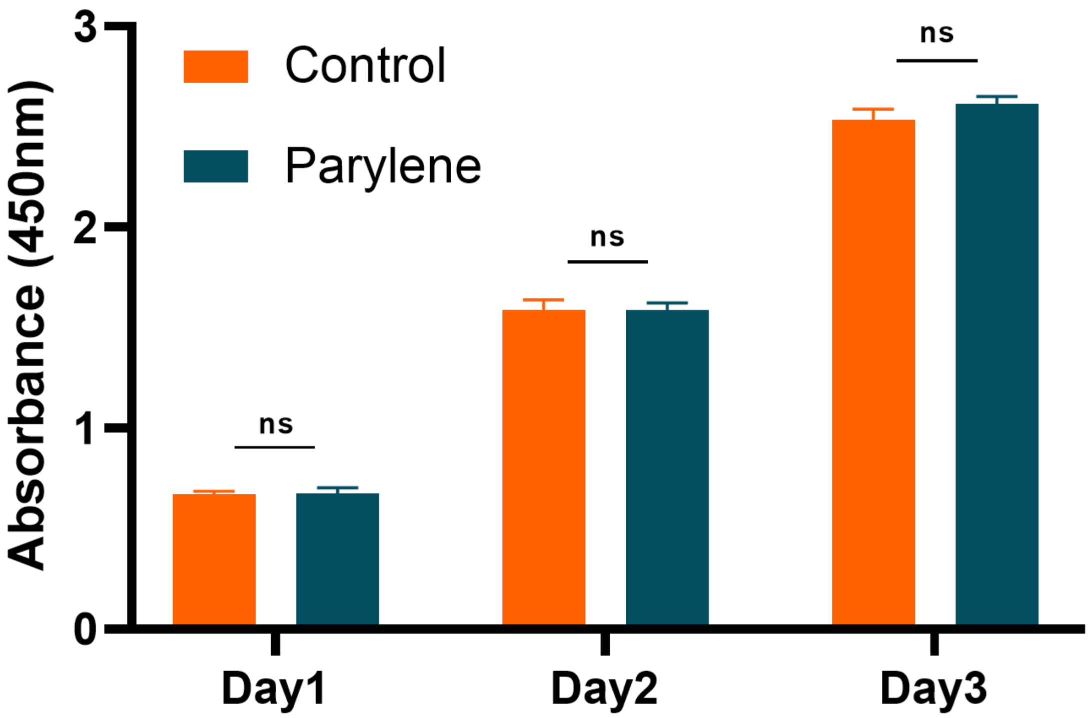

Supplementary Fig.10|The viability of L929 cells on the encapsulation film tested by the Cell Counting Kit-8 (n=3, Data are presented as mean ± SD. ns, no significant differences). Source data are provided as a Source Data file.

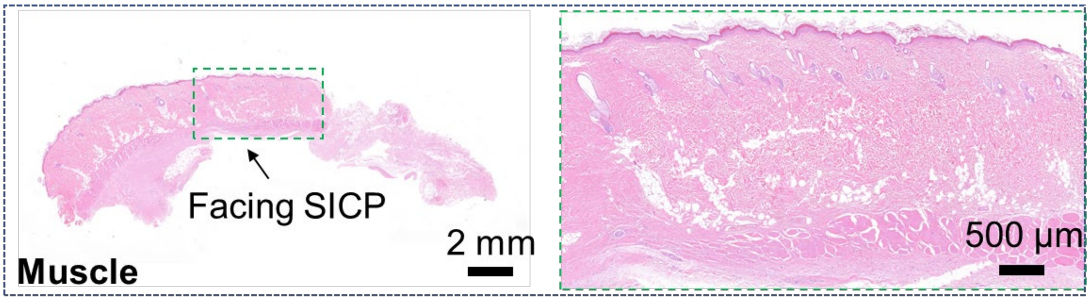

Supplementary Fig.11|. Localized tissues of the skin to deep layer muscle from the implantation location of the materials after 3 months implantation stained by Hematoxylin and Eosin (H&E).

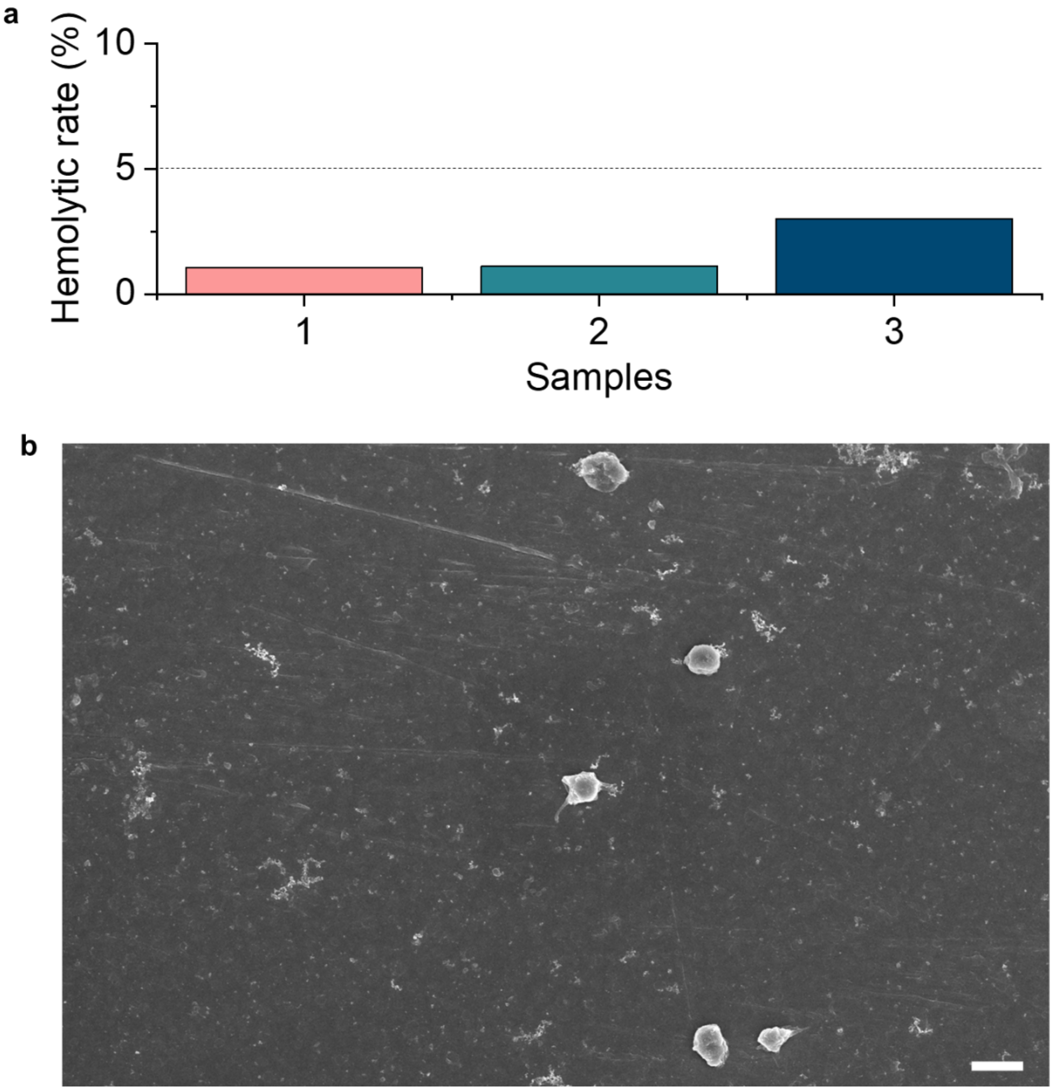

Supplementary Fig.12|a, Hemolysis (n=3) and (**b**) coagulation test for the encapsulation materials (scale bar = 2 μm). Source data are provided as a Source Data file.

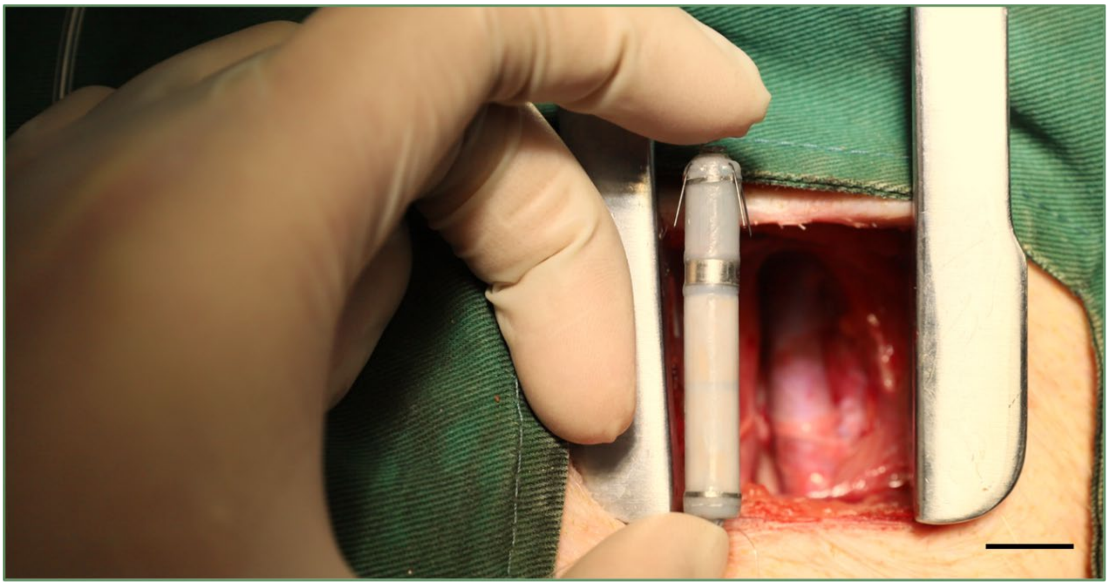

Supplementary Fig.13| A Photograph of the external jugular vein exposed with a small incision. (Scale bar = 1 cm)

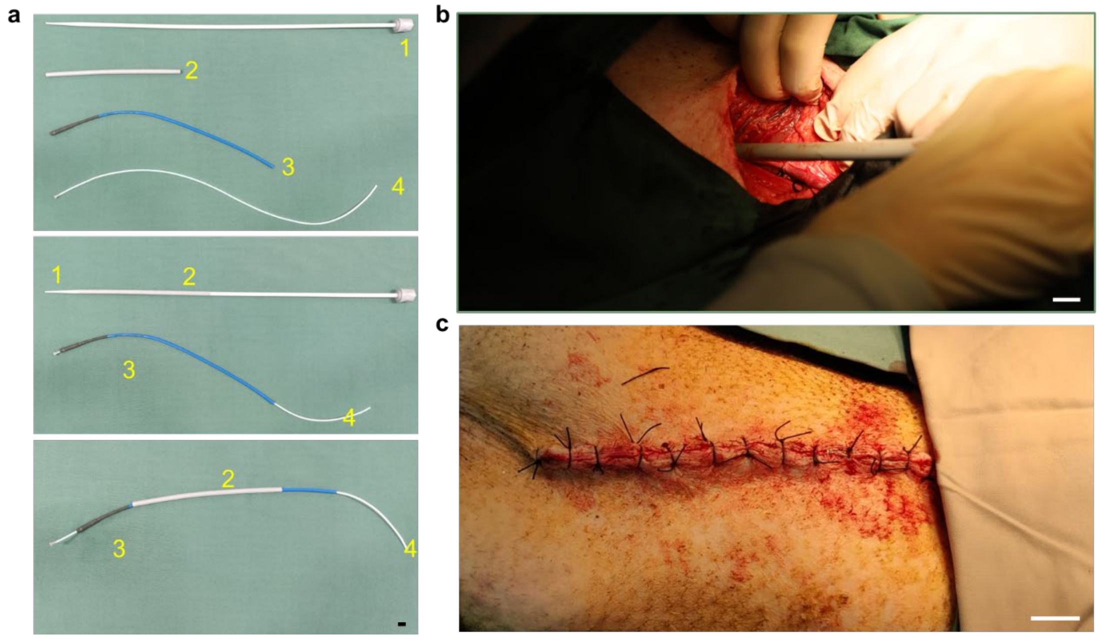

Supplementary Fig.14| Photographs of (**a**) delivery system (1: inside sheath, 2: outside sheath, 3,4: delivery catheter), (**b**) delivery catheter with SICP advancement, and (**c**) Sutured incision after device implantation. (Scale bar = 1 cm)

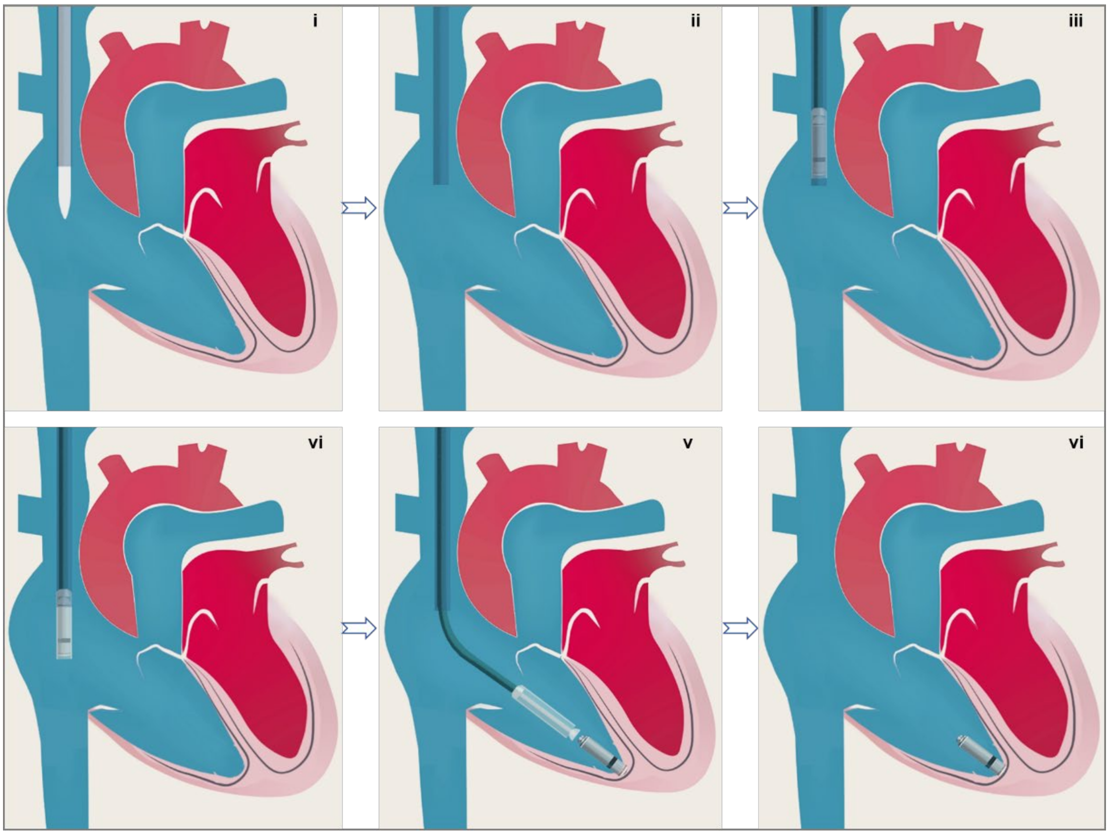

Supplementary Fig.15|The schematic diagram of SICP implantation process.

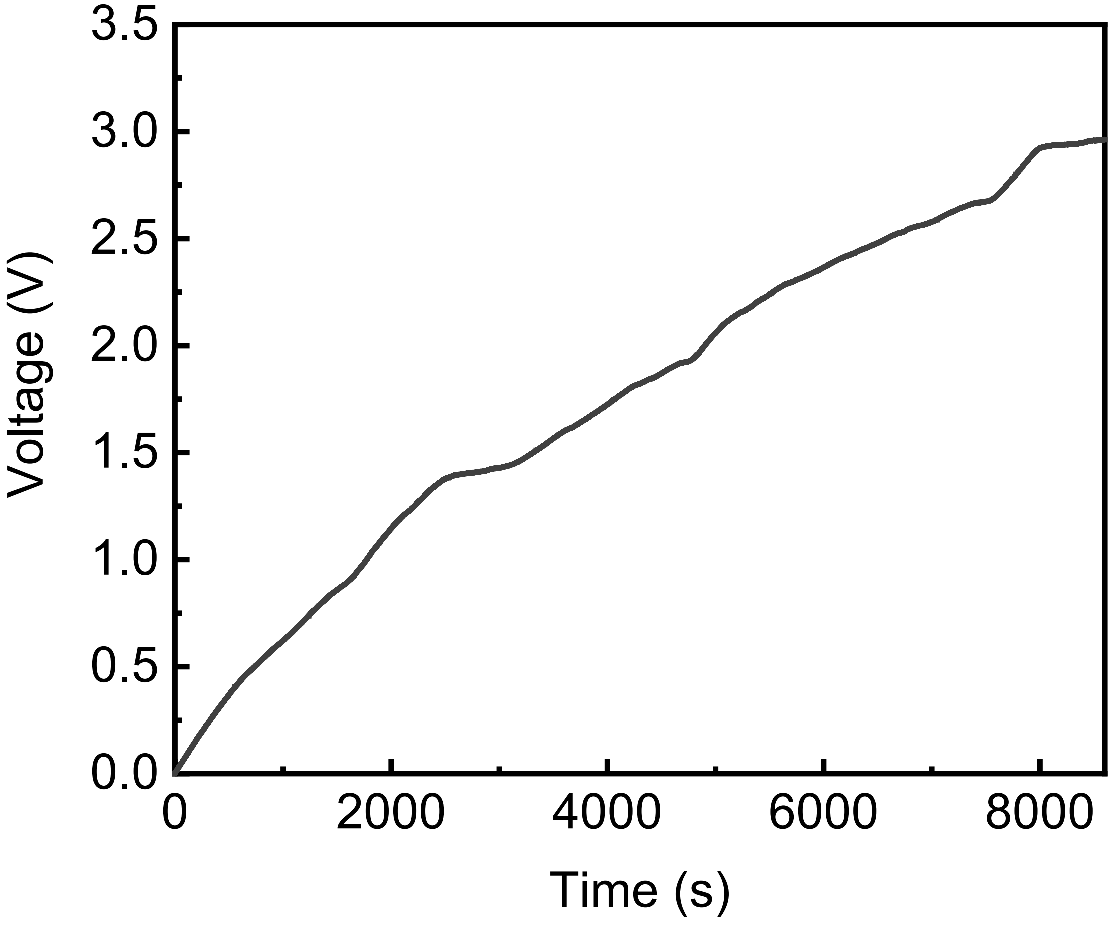

Supplementary Fig.16| The voltage of a capacitor from 0 V to 3 V within 9000 s at the same electrical output of SICP *in vivo*.

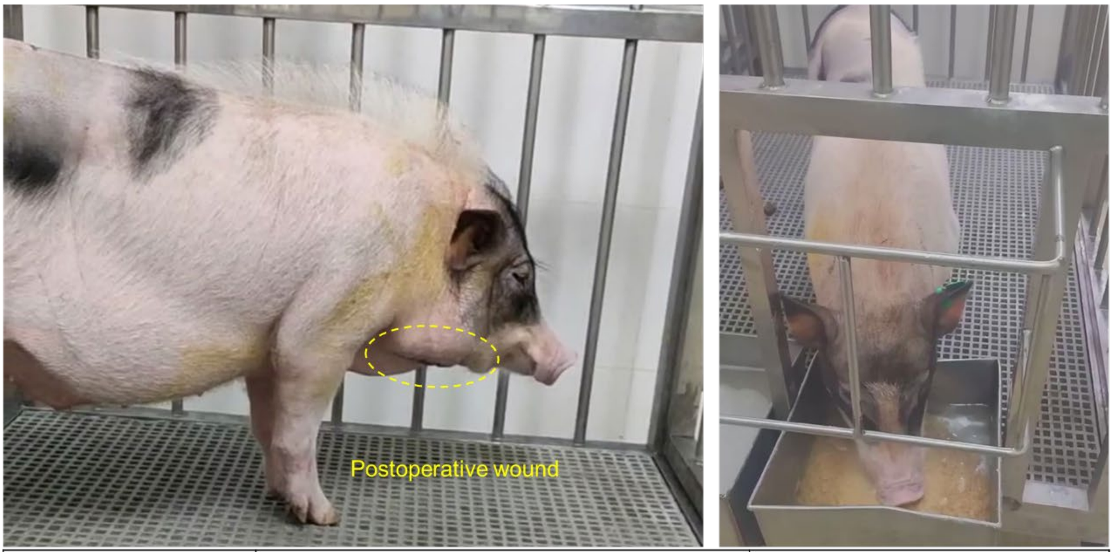

Number of animals	Experimental purposes	Experimental results
1# (a swine)	1. Induce a atrioventricular block (AVB) animal model by radiofrequency ablation<br>2. Explore the pacing efficiency for PM of SICP *in vivo*	The AVB experimental animal model was successfully constructed.<br>The PM of SICP successfully regulated the AVB animal heart rate from 33 bpm to 90 bpm.
2#-5# (four swine)	1. Evaluate the performance of the homemade introducer and dilator advancement<br>2. Energy harvesting of SICP *in vivo*<br>3. Evaluate pacing effect of SICP in acute phase	The homemade introducer and dilator advancement successfully implanted the device into the right ventricle.<br>The *in vivo* open circuit voltage and short circuit current of SICP were about 6.0 V and 0.2 μA.<br>The SICP achieved successful regulation of animals heart rate (from 90 bpm to 108 bpm) *in vivo*.
6#-8# (three swine)	Evaluate the long-term stability (energy harvesting, pacing, fixed effect and biocompatibility) of SICP *in vivo*	The SICP converts cardiac motion energy to electricity and maintains endocardial pacing function during three weeks follow-up period.

Supplementary Fig.17| Photographs of the postoperative animal and statistical analysis of animal experiments.

Reference

1. Y. Zi et al., Standards and figure-of-merits for quantifying the performance of triboelectric nanogenerators. Nature communications **6**, 8376 (2015).
2. Furrer, M. et al. VATS-guided epicardial pacemaker implantation: hand-sutured fixation of atrioventricular leads in an experimental setting. Surgical endoscopy **11**, 1167-1170 (1997).
3. Ritter, P. et al. Early performance of a miniaturized leadless cardiac pacemaker: the Micra Transcatheter Pacing Study. European heart journal **36**, 2510-2519 (2015)
