# Supplementary material for: A self-powered intracardiac pacemaker in swine model
Source: Nat Commun. 2024 Jan 13;15:507. doi: 10.1038/s41467-023-44510-6 (PMC10787765; doi:10.1038/s41467-023-44510-6)
Supplement: Supplementary file 3 — Description of additional supplementary files [file 41467_2023_44510_MOESM3_ESM.pdf]

Description of additional supplementary files

Supplementary Movie 1: The working principle of the EHU

Supplementary Movie 2: Tensile test for SICP

Supplementary Movie 3: SICP implantation under fluoroscopic guidance

Supplementary Movie 4: Minimally invasive delivery of the SICP

Supplementary Movie 5: SICP movement with heat beat after fixation under fluoroscopic guidance

Supplementary Movie 6: POM boll movement with heart beat

Supplementary Movie 7: The process of SICP regulate arrhythmia

Supplementary Movie 8: State of the experimental animal after three weeks implantation.
