# Supplementary material for: A self-powered intracardiac pacemaker in swine model
Source: Nat Commun. 2024 Jan 13;15:507. doi: 10.1038/s41467-023-44510-6 (PMC10787765; doi:10.1038/s41467-023-44510-6)
Supplement: Supplementary file 12 — Reporting Summary [file 41467_2023_44510_MOESM12_ESM.pdf]

nature portfolio

Corresponding author(s): Zhou Li

Last updated by author(s): Dec 11, 2023

Reporting Summary

Nature Portfolio wishes to improve the reproducibility of the work that we publish. This form provides structure for consistency and transparency in reporting. For further information on Nature Portfolio policies, see our Editorial Policies and the Editorial Policy Checklist.

Statistics

For all statistical analyses, confirm that the following items are present in the figure legend, table legend, main text, or Methods section.

n/a	Confirmed	
☐	☒	The exact sample size (*n*) for each experimental group/condition, given as a discrete number and unit of measurement
☐	☒	A statement on whether measurements were taken from distinct samples or whether the same sample was measured repeatedly
☐	☒	The statistical test(s) used AND whether they are one- or two-sided<br>*Only common tests should be described solely by name; describe more complex techniques in the Methods section.*
☒	☐	A description of all covariates tested
☒	☐	A description of any assumptions or corrections, such as tests of normality and adjustment for multiple comparisons
☐	☒	A full description of the statistical parameters including central tendency (e.g. means) or other basic estimates (e.g. regression coefficient) AND variation (e.g. standard deviation) or associated estimates of uncertainty (e.g. confidence intervals)
☐	☒	For null hypothesis testing, the test statistic (e.g. *F*, *t*, *r*) with confidence intervals, effect sizes, degrees of freedom and *P* value noted<br>*Give P values as exact values whenever suitable.*
☒	☐	For Bayesian analysis, information on the choice of priors and Markov chain Monte Carlo settings
☒	☐	For hierarchical and complex designs, identification of the appropriate level for tests and full reporting of outcomes
☒	☐	Estimates of effect sizes (e.g. Cohen's *d*, Pearson's *r*), indicating how they were calculated

Our web collection on statistics for biologists contains articles on many of the points above.

Software and code

Policy information about availability of computer code

Data collection	No commercial, open source nor custom code used for data collection.
Data analysis	Origin (OriginPro 2018C, OriginLab Corporation, USA) , GraphPad Prism 8.0 USA, and Excel (Microsoft Excel 2019MSO, 2209 Build 16.0.15629. 20200, Microsoft) was used for data analysis and plotting.

For manuscripts utilizing custom algorithms or software that are central to the research but not yet described in published literature, software must be made available to editors and reviewers. We strongly encourage code deposition in a community repository (e.g. GitHub). See the Nature Portfolio guidelines for submitting code & software for further information.

Data

Policy information about availability of data

All manuscripts must include a data availability statement. This statement should provide the following information, where applicable:

- Accession codes, unique identifiers, or web links for publicly available datasets
- A description of any restrictions on data availability
- For clinical datasets or third party data, please ensure that the statement adheres to our policy

Source data for the figures in this study are available from figshare with the identifier. https://doi.org/10.6084/m9.figshare.24715311

Research involving human participants, their data, or biological material

Policy information about studies with human participants or human data. See also policy information about sex, gender (identity/presentation), and sexual orientation and race, ethnicity and racism.

Reporting on sex and gender	The work did not involve human research.
Reporting on race, ethnicity, or other socially relevant groupings	The work did not involve human research.
Population characteristics	The work did not involve human research.
Recruitment	The work did not involve human research.
Ethics oversight	The work did not involve human research.

Note that full information on the approval of the study protocol must also be provided in the manuscript.

Field-specific reporting

Please select the one below that is the best fit for your research. If you are not sure, read the appropriate sections before making your selection.

[x] Life sciences   [ ] Behavioural & social sciences   [ ] Ecological, evolutionary & environmental sciences

For a reference copy of the document with all sections, see nature.com/documents/nr-reporting-summary-flat.pdf

Life sciences study design

All studies must disclose on these points even when the disclosure is negative.

Sample size	A minimum of 3 sample replicates (note that in each replicate sample) were performed. N is specified in figure legends.
Data exclusions	No data was excluded.
Replication	The sample size in cell experimental groups were ≥ 3 to ensure replicability. From the measured results, the data showed high similarity within the same testing group, and the replicability was good in each group
Randomization	Swine and rats were randomly allocated to treatment groups. Image acquisition was randomized for immunofluorescence.
Blinding	No blinding measures were deliberately taken during data collection and analysis, and all the data were processed by multiple authors.

Reporting for specific materials, systems and methods

We require information from authors about some types of materials, experimental systems and methods used in many studies. Here, indicate whether each material, system or method listed is relevant to your study. If you are not sure if a list item applies to your research, read the appropriate section before selecting a response.

Materials & experimental systems

n/a	Involved in the study	
[x]	[ ]	Antibodies
[ ]	[x]	Eukaryotic cell lines
[x]	[ ]	Palaeontology and archaeology
[ ]	[x]	Animals and other organisms
[x]	[ ]	Clinical data
[x]	[ ]	Dual use research of concern
[x]	[ ]	Plants

Methods

n/a	Involved in the study	
[x]	[ ]	ChIP-seq
[x]	[ ]	Flow cytometry
[x]	[ ]	MRI-based neuroimaging

Eukaryotic cell lines

Policy information about cell lines and Sex and Gender in Research

Cell line source(s)	The L929 cells (fibroblasts, GNM28) were acquired from the Cell Bank of the Chinese Academy of Sciences in Beijing, China.
Authentication	No authentication as such was performed. However, at least three biological replicates were preformed for each donor cells to confirm the consistency of the cell lines.
Mycoplasma contamination	All cell lines were negative for mycoplasma.
Commonly misidentified lines (See ICLAC register)	No commonly misidentified lines were used in the work.

Animals and other research organisms

Policy information about studies involving animals; ARRIVE guidelines recommended for reporting animal research, and Sex and Gender in Research

Laboratory animals	Adult swine (age: 1.2-2.0 years; weight: 50-65 kg; n=8, female=5, male=3) were acquired from State Key Laboratory of Cardiovascular Disease and Fuwai Hospital. SD rats (100 g, Male, n=10) were acquired from Beijing Vital River Laboratory Animal Technology Co., Ltd., China.
Wild animals	This study did not involve wild animals.
Reporting on sex	Male and Female.
Field-collected samples	This study did not involve samples collected from the field.
Ethics oversight	All experimental process was strictly in line with the institutional and national guidelines for the care and use of laboratory animals and the study protocol was reviewed and approved by the Ethical Committee of Animal Experimental Center in State Key Laboratory of Cardiovascular Disease and Fuwai Hospital (0103-1-1-ZX(Y)-3).

Note that full information on the approval of the study protocol must also be provided in the manuscript.
